# INTERVENTION FIDELITY IN THE FAMILY-CENTERED FUNCTION-FOCUSED CARE INTERVENTION

**DOI:** 10.1093/geroni/igac059.384

**Published:** 2022-12-20

**Authors:** Barbara Resnick

**Affiliations:** University of Maryland, Baltimore, Maryland, United States

## Abstract

This session will provide a description of the treatment fidelity (TF) plan from the Family-centered Function-focused Care (Fam-FFC) trial. Components of the TF plan, measures, procedures for implementation, and findings will be presented, and discussed within the context of the COVID-19 pandemic. The components of the Fam-FFC TF plan and results include: 1) Delivery based on completion of the steps in Fam-FFC ; 2) Receipt based on evidence of Staff knowledge of Fam-FFC (percentage of nursing staff that demonstrated test scores above 80%); 3) Enactment based on achievement of goals using the Goal Attainment Scale ; completion of the Fam-Path Audit of bedside goals and treatment plans, post-acute follow-up and plan update ; and evidence of Fam-FFC based on the Fam-FFC Behavior Checklist (80% staff performance of Fam-FFC). The TF plan demonstrated evidence of delivery, receipt and enactment of study activities. Findings will be used to develop an implementation trial.

